# An unusual cause of alveolar hemorrhage post hematopoietic stem cell transplantation: A case report

**DOI:** 10.1186/1471-2407-6-87

**Published:** 2006-04-07

**Authors:** Sachin Gupta, Amit Jain, Tina V Fanning, Daniel R Couriel, Carlos A Jimenez, Georgie A Eapen

**Affiliations:** 1 Department of Pulmonary Medicine, The University of Texas M.D. Anderson Cancer Center, Houston, Texas, USA; 2 Department of Pathology, The University of Texas M.D. Anderson Cancer Center, Houston, Texas, USA; 3 Department of Blood and Marrow Transplantation, The University of Texas M.D. Anderson Cancer Center, Houston, Texas, USA

## Abstract

**Background:**

Hematopoietic stem cell transplantation is being increasingly used in cancer therapy. Diffuse alveolar hemorrhage, an early complication of stem cell transplant, results from bacterial, viral and fungal infections, coagulopathy, and engraftment syndrome, or can be idiopathic. Diffuse alveolar hemorrhage associated with Strongyloides stercoralis hyperinfection in stem cell transplant patients has been rarely reported.

**Case presentation:**

We describe an unusual cause of alveolar hemorrhage post hematopoietic stem cell transplant due to Strongyloides hyperinfection. Therapy with parenteral ivermectin and thiabendazole was initiated but the patient deteriorated and died of respiratory failure and septic shock.

**Conclusion:**

Strongyloides stercoralis hyperinfection is an unusual cause of alveolar hemorrhage early after hematopoietic stem cell transplant with very high mortality.

## Background

Hematopoietic Stem Cell Transplantation (HSCT) is being widely used as an adjunct to conventional chemoradiotherapy for many hematologic and solid malignancies. Diffuse alveolar hemorrhage (DAH) in this setting can occur due to bacterial, viral and fungal infections, coagulopathy, cardiac causes, graft versus host disease (GVHD), drug and radiation toxicity, or it can be idiopathic. DAH associated with Strongyloides stercoralis hyperinfection in stem cell transplant patients has been rarely reported. We present an interesting case of Strongyloides hyperinfection syndrome manifesting as alveolar hemorrhage and gram negative septicemia. A brief review of literature on this topic is also presented.

## Case presentation

A 52-year old Puerto Rican male presented with acute onset nausea, vomiting, and abdominal pain 24 days after autologous Hematopoietic Stem Cell Transplantation (HSCT) for recurrent stage advanced follicular lymphoma.

The lymphoma was diagnosed 7 years prior to transplant, and was treated with different chemotherapy agents, including Cyclophosphamide, doxorubicin, vincristine, prednisone (CHOP regimen) and rituximab. Following relapse, he underwent autologous HSCT following a conditioning regimen with carmustine (BCNU), etoposide, adriamycin, and melphalan (BEAM). Neutrophil engraftment occurred on day nine. Antimicrobial prophylaxis included valacyclovir, fluconazole, levofloxacin, and trimethoprim-sulfamethoxazole. The patient was not taking steroids or other immunosuppressive medicines at this time.

Past medical and social history was unremarkable. There was no significant occupational exposure history. Patient was noted to have elevated eosinophil count (17% eosinophils; absolute count 0.970 × 10^9^/L) on several occasions, three months prior to the transplant.

On physical examination, his temperature was 37.7°C, heart rate 85/min, respiratory rate 16/min, blood pressure 110/66 mm Hg, and pulse oximetry 96% on room air. The respiratory, cardiovascular and abdominal examination was normal.

The white blood cell count was 7.2 × 10^9^/L (67% neutrophils, 18% bands, 5% lymphocytes, 7% monocytes, 2% metamyelocytes, and 1% eosinophils), hemoglobin 11.6 g/dL, platelets 25 × 10^9^/L. Serum chemistry was normal.

**Figure 1:**
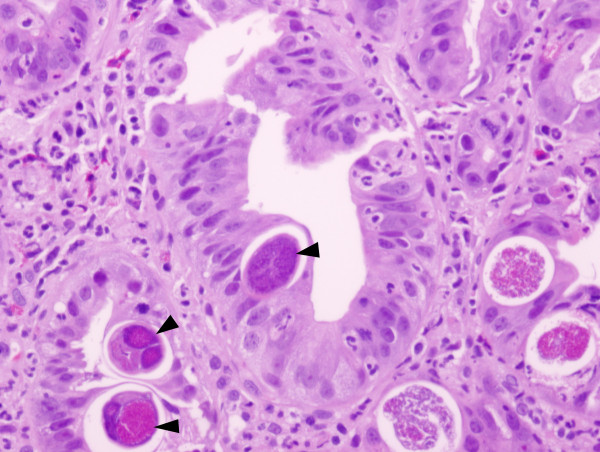
Duodenal biopsy showing Strongyloides stercoralis within the glandular cells of the mucosa (marked with solid arrows). The underlying lamina propria showed abundant neutrophils, eosinophils and chronic inflammatory cells. Hematoxylin and eosin stain; 200×.

The patient underwent esophagogastroduodenoscopy (EGD) with duodenal biopsy for persistent nausea and vomiting. The biopsy showed active inflammation, larval forms of Strongyloides stercoralis (Figure 1) and stromal cells with rare cytomegalovirus inclusions. Stool microscopy for ova and parasites was negative. Intra venous immune globulin (IVIG), Ganciclovir, Foscarnet, Ivermectin 15 mg daily and thiabendazole 3 gm daily were started with relief of symptoms within three days.

Ten days later, the patient developed dyspnea, fever, and hypoxia. Bilateral crackles were noted on lung auscultation. His clinical status deteriorated rapidly developing hypotension and respiratory failure.

**Figure 2:**
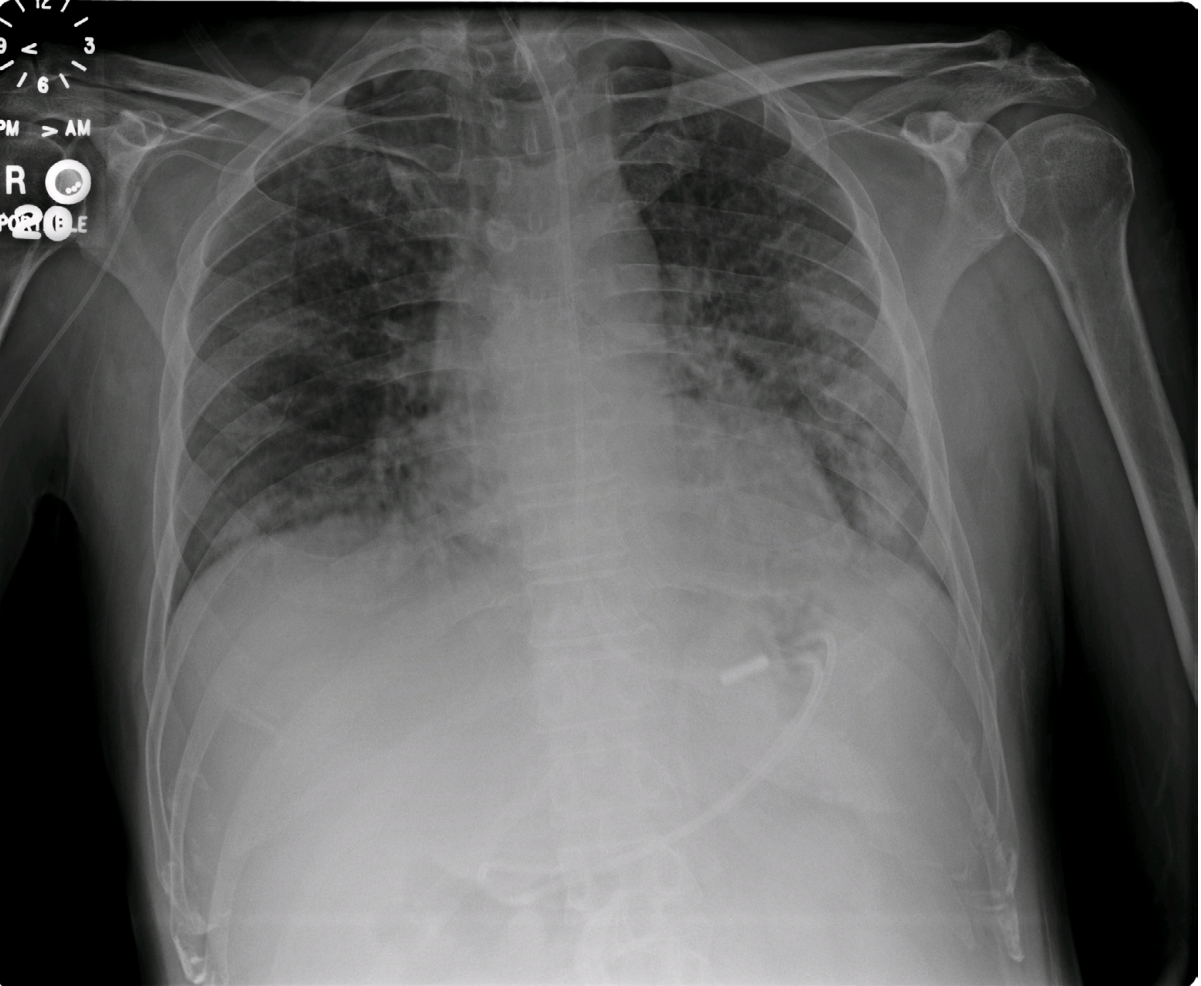
Antero-posterior view of the chest x-ray, showing bilateral diffuse alveolar opacities.

While on mechanical ventilation with 100% oxygen, pH was 7.45; pCO_2 _33 mm Hg and pO_2 _120 mm Hg. Thrombocytopenia, anemia, and neutrophilia were noted (Table 1). The chest x-ray showed diffuse airspace disease (Figure 2). Echocardiogram was normal. Therapy with Meropenem, gentamicin, and vancomycin was initiated. Blood cultures later grew Klebsiella pneumoniae. Vancomycin was discontinued, while meropenem and gentamicin were continued with clinical improvement leading to extubation in four days.

**Table 1:** Laboratory data at the onset of respiratory distress.

Hemoglobin (gm/dl)	7.8
Hematocrit (%)	22.9
WBC (*10^9^/L)	8.4
Neutrophils (%)	92
Lymphocytes (%)	6
Monocytes (%)	2
Eosinophils (%)	0
Platelet (*10^9^/L)	36
BUN (mg/dl)	28
Creatinine (mg/dl)	0.8
PT (seconds)	12.9
PTT (seconds)	22.9
INR	1.31
HIV	Negative
HBV	Negative
HCV	Negative
RPR	Negative

Ten days later, he was reintubated for recurrent respiratory failure and ventilatory support using BiLevel mode was provided. Bronchoscopy with bronchoalveolar lavage (BAL) was suggestive of diffuse alveolar hemorrhage (DAH). High dose parenteral steroids were initiated.

**Figure 3:**
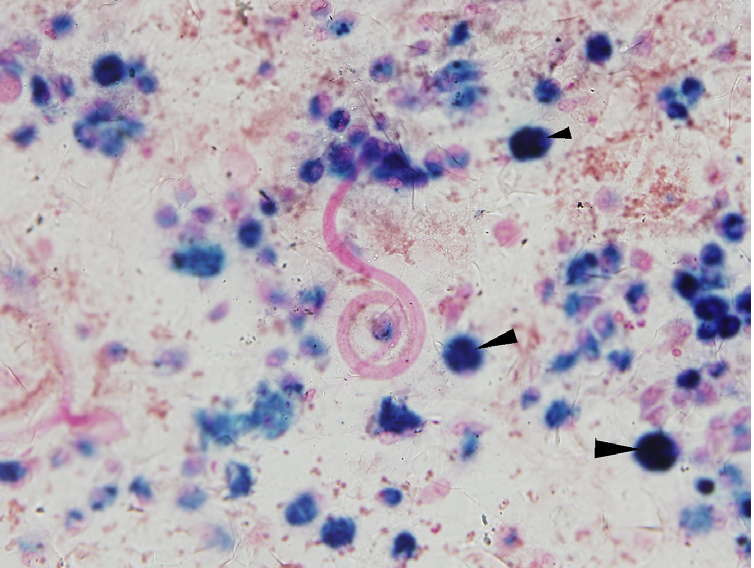
Bronchoalveolar lavage showing larval forms of Strongyloides (stained pink) in a background of many hemosiderin laden macrophages (stained blue and marked with solid arrows). Prussian blue stain for iron; 200×. Stains for fungus and Pneumocystis were negative.

The BAL fluid showed multiple larval forms of Strongyloides stercoralis (Figure 3), 90% hemosiderin laden macrophages and later grew Klebsiella pneumoniae. The steroids were discontinued and patient was started on intravenous Ivermectin.

His pulmonary status continued to deteriorate. The patient developed pneumothorax and subcutaneous emphysema, and died of progressive respiratory failure and septic shock 14 days later.

## Discussion

Diffuse alveolar hemorrhage syndrome is characterized by respiratory compromise, diffuse alveolar infiltrates and progressively bloody BAL in a post-HSCT setting. The pathogenesis of DAH syndrome is unknown. The causes of alveolar hemorrhage in post-transplant patients include bacterial, fungal and viral pneumonia, coagulopathy, cardiac dysfunction, radiation or drug toxicity, engraftment syndrome, and idiopathic DAH syndrome. The intestinal nematode, Strongyloides stercoralis is rarely reported in association with alveolar hemorrhage in HSCT recipients [1,2].

Strongyloidiasis is endemic in parts of Asia, South America, Europe, and Southeastern United States [2]. The infection is usually asymptomatic and confined to the intestinal tract. After penetrating the skin, the filariform larva enters the blood stream and is transported to the lungs. The life cycle involves migration up the respiratory tract, followed by swallowing into the gastrointestinal tract. Maturation and reproduction in the small intestine results in the formation of rhabditiform and filariform larvae. Filariform larvae penetrate the intestinal wall or perianal skin causing autoinfection. When cell mediated immunity is suppressed, the parasite proliferates and develops a large burden of the infective filariform larvae which penetrate the intestinal mucosa and disseminate through the blood stream resulting in hyperinfection. During penetration of the intestinal epithelium, enteral bacteria (usually gram negative coliforms) disseminate systemically. This can lead to gram negative sepsis, a common and potentially serious complication of Strongyloides hyperinfection syndrome. Other pulmonary complications that can occur include lung abscess and ARDS [3].

Eosinopenia post HSCT may be important in the genesis of hyperinfection as eosinophils are a fundamental component of the immune response to parasitic infections. Mechanical destruction of pulmonary capillaries by the larvae, cytotoxic chemokines released by the parasites, influx of inflammatory cells and severe endothelial inflammation that occurs in the periengraftment period may all contribute to the resultant alveolar hemorrhage. During hyperinfection, pathologic findings include a mixed cellular infiltrate of lymphocytes, macrophages, and neutrophils within the alveolar septal walls associated with alveolar, interstitial, and intra-vascular larvae [4].

Strongyloides hyperinfection after solid organ transplantation usually coincides with steroid use for graft rejection and manifests between 1–3 months post-transplant. Profound immunosuppression during HSCT may be responsible for its earlier presentation post HSCT.

Strongyloides hyperinfection has a mortality exceeding 80% [5] and eradication of strongyloides prior to HSCT has been reported to improve outcome [6]. Screening of patients from endemic and high prevalence regions coupled with appropriate treatment prior to HSCT could avoid a potentially fatal outcome. The optimal screening strategy is controversial. Seventy five percent of individuals with strongyloides infection manifest eosinophilia [7]. The prevalence of eosinophilia in immunocompromised patients with chronic strongyloidiasis is less than 20%, making this a poor screening test. Our patient did have persistent eosinophilia prior to the transplant. Examination of >3 stool samples for direct identification of Strongyloides larval forms has a sensitivity of 60–70%, and this may even be higher among immunocompromised patients. A positive ELISA test is accurate in 97% of the cases with a negative predictive value of 95% in patients from endemic areas [8]. Given the dreadful outcomes associated with strongyloides hyperinfection following HSCT, screening with peripheral eosinophil counts and a strongyloides ELISA along with stool examination for confirmation may be warranted prior to HSCT. Significant eosinophilia before transplant may mandate workup to rule out infections associated with it.

Ivermectin (150–200 μg/kg/d) is the drug of choice, the alternative being thiabendazole (50 mg/kg/d, maximum 3.0 g/d) [9]. There are currently no guidelines for the treatment of strongyloides hyperinfection in the immunosuppressed patients. Whether combination treatment is better than ivermectin or thiabendazole alone is not known. High dose steroids can exacerbate the Strongyloides hyperinfection syndrome.

## Conclusion

Strongyloides stercoralis hyperinfection is a possible cause of alveolar hemorrhage early after HSCT. A high index of suspicion, pre-transplant screening, and aggressive treatment in patients from endemic areas may improve outcome. Further studies are needed to determine the role of parenteral Ivermectin and combination treatment with Ivermectin and Thiabendazole for treatment of disseminated strongyloidiasis.

## Abbreviations

ARDS = Acute Respiratory Distress Syndrome

BAL = Bronchoalveolar Lavage

DAH = Diffuse Alveolar Hemorrhage

GVHD = Graft Versus Host Disease

HSCT = Hematopoietic Stem Cell Transplantation

IVIG = Intra Venous Immune Globulin

## Competing interests

All authors declare that there are no competing interests (financial or non-financial) and have nothing to declare.

## Authors' contributions

SG prepared the manuscript, reviewed the literature and edited the report. AJ did review of literature, was involved in writing the manuscript, and editing the figures. TVF participated in patient care, described the pathology reports, and prepared the figures. DRC was involved in the care of patient and performed the HSCT. CAJ was involved in the care of patient and performed the bronchoscopy, and helped in manuscript preparation. GAE provided expert guidance throughout the preparation of the manuscript, reviewed and edited the report. All authors have read and approved the final manuscript.

## Pre-publication history

The pre-publication history for this paper can be accessed here:


